# Muscle-brain crosstalk in sarcopenia and cognitive impairment: endocrine signaling, metabolic regulation, and exercise modulation

**DOI:** 10.3389/fendo.2026.1929487

**Published:** 2026-09-16

**Authors:** Haodong Tian, Li Huang, Haowei Liu, Hansen Li, Yanxia Zhao

**Affiliations:** 1 College of Physical Education, Southwest University, Chongqing, China; 2 Faculty of Psychology, Southwest University, Chongqing, China; 3 School of Sports Training, Chengdu Sport University, Chengdu, Sichuan, China; 4 School of Physical Education, Sichuan Agricultural University, Ya’an, China; 5 College of Physical Education, Chongqing University, Chongqing, China

**Keywords:** cognitive impairment, endocrine, exercise, metabolic regulation, muscle-brain crosstalk, sarcopenia

## Abstract

Aging is accompanied by progressive changes in endocrine signaling, metabolic regulation, and inter-organ communication that may contribute to functional decline. Sarcopenia and cognitive impairment frequently coexist in older adults, raising the possibility that their association involves disruption of communication between skeletal muscle and the brain in addition to shared age-related processes. This narrative review synthesizes clinical and mechanistic evidence on muscle–brain crosstalk in sarcopenia and cognitive impairment, with emphasis on endocrine and metabolic mechanisms and their modulation by exercise. Clinical evidence consistently links sarcopenia with cognitive impairment, while muscle strength and physical performance appear more closely related to cognitive function than muscle mass alone. Potential mechanisms include myokine and exerkine signaling, inflammaging and immune–endocrine dysregulation, mitochondrial dysfunction and oxidative stress, insulin resistance and glucose dysregulation, and lactate-mediated metabolic signaling. Exercise provides a dynamic physiological context in which several components of muscle–brain communication can be modified, including inflammatory, metabolic, neurotrophic, and lactate-related responses. However, direct evidence establishing complete signaling pathways in humans remains limited. Future studies integrating muscle and cognitive phenotypes with circulating biomarkers, metabolic measures, and neuroimaging are needed to clarify the clinical relevance of muscle–brain crosstalk in individuals with coexisting sarcopenia and cognitive impairment.

## 1 Introduction

Population aging is an inevitable outcome of socioeconomic development. However, aging itself is not the core threat to public health. The pace of aging is relatively constant across individuals and over time, and increases in life expectancy are not mainly due to a slowing of aging, but rather to continuous improvements in health conditions (1). In fact, the increased risk of age-related diseases is the real source of health challenges and healthcare system burden. In industrialized countries worldwide, approximately one-third of healthcare expenditure is devoted to disease treatment and health management for citizens aged over 70 years (2). For this reason, “healthy aging” has become a major goal of aging societies, with the management of geriatric diseases serving as the crucial approach (3). Nevertheless, addressing these complex and interconnected conditions remains challenging, as they are often rooted in age-related decline across multiple organ systems (4). Exploring the inter-organ physiological crosstalk underlying these diseases is therefore essential for understanding comorbidity and developing therapeutic strategies.

Sarcopenia, often described as one of the “Giants of Geriatrics” (5), is a progressive age-related skeletal muscle disorder characterized by declines in muscle mass, strength, and physical performance. In 2016, it was officially recognized as a disease entity and assigned the ICD-10-CM code M62.84 (6). Beyond its direct effects on mobility, physical function, and independence (7), sarcopenia is increasingly recognized as a systemic condition associated with a wide range of adverse outcomes, including metabolic disorders (8–10), osteoporosis (11), frailty (12), cognitive impairment (13), and increased mortality. Similar to sarcopenia, cognitive impairment is another major age-related condition that affects a substantial proportion of older adults worldwide. Cognitive decline is almost inevitable during aging and usually becomes more pronounced with increasing age (14). Among older adults, declines in memory, attention, executive function, language, and visuospatial abilities contribute to loss of independence, reduced quality of life, and increased risks of adverse health events (15, 16). As age advances, the risk of coexisting sarcopenia and cognitive impairment increases, making these two conditions major public health challenges in aging society and highlighting the need to better understand the biological mechanisms that link them.

Accumulating evidence suggests a substantial bidirectional relationship between sarcopenia and cognitive impairment (17–19). This association may be partly explained by the complex interaction between skeletal muscle and the brain during aging. As highly energy-demanding organs, both tissues rely on adequate energy supply, efficient mitochondrial function, and tightly regulated metabolic processes to maintain structural and functional integrity (20–24). With advancing age, shared biological alterations may create a context in which muscle decline and cognitive decline reinforce each other. Skeletal muscle and the brain communicate continuously through endocrine, immune, neural, and metabolic pathways (25).

Skeletal muscle is now recognized as an endocrine organ rather than merely an effector of movement and energy expenditure (26, 27). Through the secretion of myokines, metabolites, and extracellular vesicles, skeletal muscle can influence brain structure and function by transmitting endocrine and metabolic signals across the blood-brain barrier (28). Conversely, the brain regulates motor activity and muscle homeostasis through integrated neuroendocrine and behavioral pathways. Notably, many of the mediators involved in this bidirectional communication are highly responsive to muscle contraction and neural activity. From a physiological perspective, exercise represents a neurally coordinated form of skeletal muscle activity, and repeated exercise induces adaptive responses involving both the neuromuscular system and systemic signaling networks. These features make exercise particularly relevant to the study of muscle–brain crosstalk and are consistent with its established role in the non-pharmacological management of sarcopenia and cognitive impairment (29, 30). Against this background, exercise can provide a dynamic physiological context for exploring potentially beneficial endocrine-metabolic communication between skeletal muscle and the brain. Characterizing these responses may help clarify how muscle–brain crosstalk is altered in sarcopenia and cognitive impairment and whether it represents a potential target for intervention, particularly when the two conditions coexist.

This review focuses on the endocrine and metabolic dysregulation underlying their relationship within the framework of muscle–brain crosstalk. Particular attention is given to myokine and exerkine signaling, inflammatory and immune dysregulation, mitochondrial dysfunction and oxidative stress, insulin and glucose dysregulation, and lactate signaling. As exercise is an important strategy for managing both sarcopenia and cognitive impairment, its role is further considered from the perspective of modulating muscle–brain communication rather than its broader health benefits.

## 2 Review approach

This article is a narrative review that synthesizes current evidence on muscle–brain crosstalk in sarcopenia and cognitive impairment, with particular attention to endocrine signaling, metabolic regulation, and their modulation by exercise. Relevant literature published up to June 15, 2026, was identified through searches of PubMed, Web of Science, Embase, the Cochrane Library, and the China National Knowledge Infrastructure (CNKI). The initial search was organized around broad combinations of terms related to *sarcopenia, skeletal muscle, muscle strength and physical performance; cognitive impairment, cognitive decline, cognitive function, dementia; and muscle–brain crosstalk, muscle–brain communication, inter-organ communication, endocrine signaling, and metabolic regulation*. Exercise and physical activity terms were also included where relevant to muscle–brain communication. As specific biological pathways emerged during the narrative synthesis, additional targeted searches were conducted to examine the underlying mechanisms and relevant disease contexts in greater depth.

Given the mechanistic focus of the review, human clinical and observational evidence was prioritized when available, while experimental studies were used to clarify biological pathways that remain difficult to establish directly in humans. Systematic reviews and meta-analyses were also considered when relevant to the consistency of clinical evidence. Studies were included in the narrative synthesis when they informed the clinical relationship between sarcopenia and cognition, the biological basis of muscle–brain communication, or mechanistic pathways identified during the synthesis. Experimental studies were included when they provided mechanistic evidence not readily available from human studies. The search was intended to support a focused narrative synthesis rather than an exhaustive systematic review, and no formal study-level risk-of-bias assessment was performed. Throughout the review, human evidence is distinguished from preclinical mechanistic evidence and biological inference.

## 3 Clinical links between sarcopenia and cognitive impairment

The clinical evidence summarized in this section was derived from studies using established operational definitions of sarcopenia, including consensus-based criteria such as those proposed by the European Working Group on Sarcopenia in Older People (EWGSOP) (31) and the Asian Working Group for Sarcopenia (AWGS) (32). Although these frameworks differ in specific diagnostic algorithms and population-specific cut-off values, they represent accepted operationalizations of the same clinical construct. In this review, cognitive impairment is used as a broad clinical term encompassing different stages of cognitive dysfunction, particularly mild cognitive impairment and dementia (15, 33). Studies examining clinically or screening-defined cognitive impairment are discussed separately from those assessing cognitive performance or specific cognitive domains. This distinction is maintained throughout the following sections when interpreting the clinical relationship between muscle and cognitive health.

### 3.1 Sarcopenia and cognitive impairment

A growing body of evidence supports a close association between sarcopenia and cognitive impairment in older adults (**Figure 1**). A meta-analysis reported that individuals with sarcopenia had a significantly higher risk of cognitive impairment than those without sarcopenia (adjusted OR = 2.2, 95% CI: 1.2-4.2), and this relationship appeared to be independent of ethnicity, sex, and cognitive assessment methods (34). Consistent findings have been reported in several cross-sectional studies. A study of older Korean women found that the odds of cognitive impairment were approximately two-fold higher among individuals with possible sarcopenia and five-fold higher among those with sarcopenia compared with non-sarcopenic controls (35). Similar associations have also been observed in large cohorts of community-dwelling and hospitalized older adults. Especially in women, both the lower muscle mass and fat mass represent risk factors of cognitive decline (36, 37).

**Figure 1:**
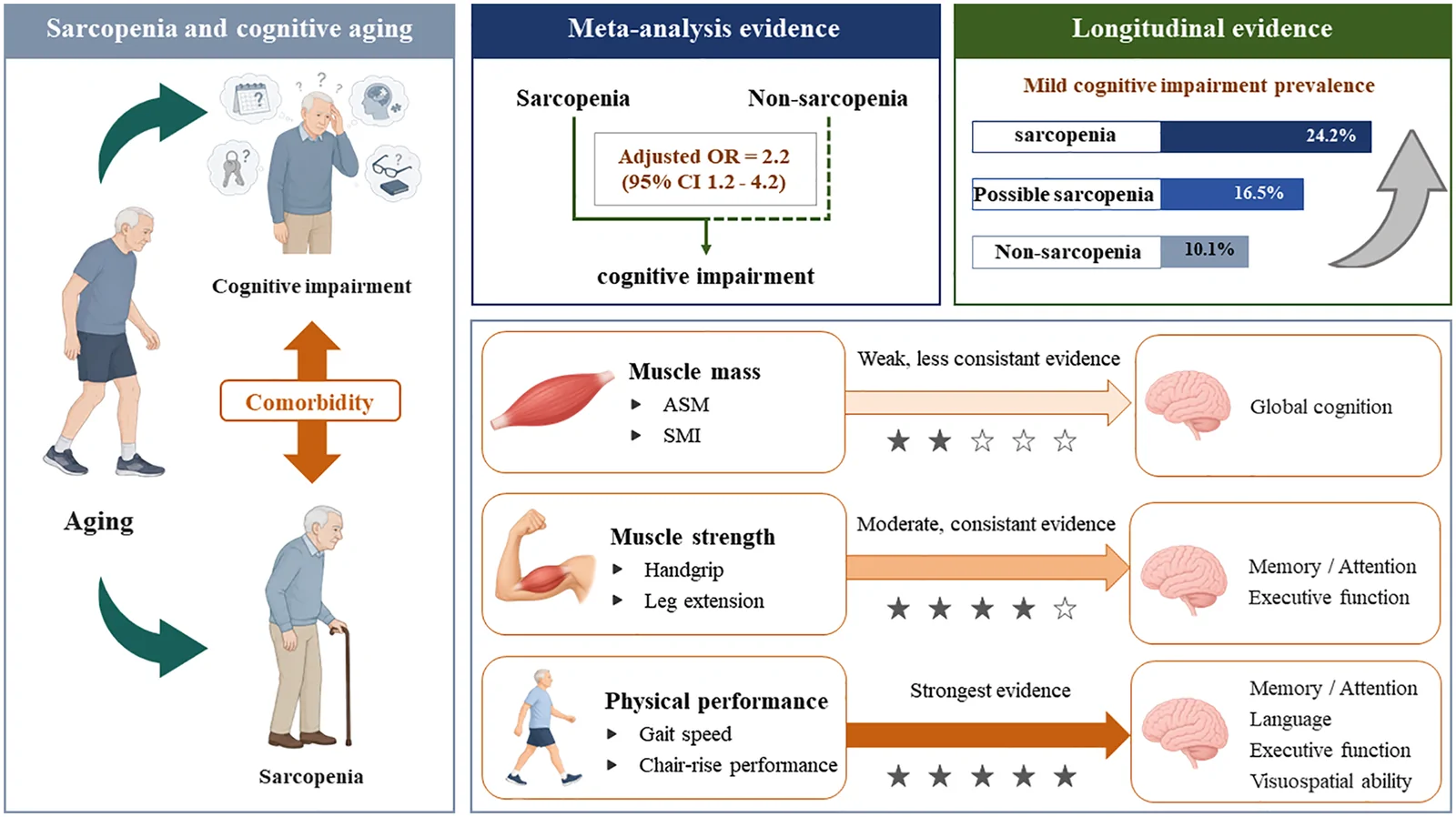
Clinical evidence linking sarcopenia and cognitive impairment during aging. ASM, Appendicular skeletal muscle mass; SMI, Skeletal muscle mass index.

Longitudinal evidence provides stronger support for the relationship between sarcopenia and cognitive decline. In a large-scale prospective study based on the China Health and Retirement Longitudinal Study (CHARLS), involving 5,715 participants, progression from non-sarcopenia to sarcopenia was accompanied by significant declines in orientation, calculation, memory, and visuospatial abilities. Moreover, the prevalence of mild cognitive impairment increased progressively across non-sarcopenic (10.1%), possible sarcopenic (16.5%), and sarcopenic (24.2%) groups (19). Nevertheless, some studies have reported inconsistent findings. One investigation involving 3,025 women aged over 75 years found no significant association between sarcopenia and cognitive impairment regardless of the diagnostic criteria applied (38). Another study suggested that the relationship between sarcopenia and cognitive impairment was not evident among adults aged 60-69 years but became significant after the age of 70 years (39). Collectively, these findings indicate that the association between sarcopenia and cognitive impairment may strengthen with increasing age, although heterogeneity remains across populations and study designs.

### 3.2 Muscle strength, physical performance, and cognitive function

Among older adults, increasing attention has been paid to the cognitive relevance of muscle strength and physical performance, two key components of sarcopenia phenotypes. Notably, several studies suggest that these functional indicators may be more closely related to cognitive health than muscle mass itself (**Figure 1**). Cross-sectional studies have consistently demonstrated positive associations between muscle strength and cognitive performance. In a study of Japanese adults aged 85 years and older, both handgrip strength and knee extensor strength were significantly associated with higher cognitive function after adjustment for potential confounders (40). Similar findings have been reported in larger cohorts, where greater quadriceps strength was linked to better visuospatial ability, processing speed, and memory performance (41). Furthermore, longitudinal investigations indicated that slower declines in handgrip strength are associated with better preservation of attention, memory, and executive function in later life (42–44). However, evidence on the temporal direction of this relationship remains mixed. In a 4-year follow-up study of adults aged 85 years, better baseline cognitive performance predicted a slower decline in handgrip strength, whereas baseline handgrip strength did not consistently predict subsequent cognitive decline (45). This inconsistency may partly reflect differences in study design, cognitive assessment methods, muscle strength measurements, and participant age. Nevertheless, the overall body of evidence supports a close association between muscle strength and cognitive function in later life.

Physical performance appears to exhibit an even stronger relationship with cognition (**Figure 1**). Because tasks such as gait and chair-rise performance require the integration of motor, sensory, and cognitive processes, they may better reflect muscle-brain interactions. In a large community-based study of approximately 4,000 Chinese adults, individuals with cognitive impairment demonstrated significantly slower gait speed and poorer chair-rise performance than cognitively healthy controls (46). Subsequent investigations incorporating more detailed gait parameters, including speed, rhythm, and variability, reported consistent associations across multiple cognitive domains, including memory, executive function, attention, and language ability (47). In addition to gait-related measures, poorer performance on the Timed Up-and-Go and Five Times Sit-to-Stand tests has also been associated with cognitive impairment. This association remained significant after adjustment for age, sex, and BMI (48). In summary, current evidence suggests that muscle strength and physical performance show more robust and consistent associations with cognitive function than muscle mass alone, supporting the concept of a close functional linkage between skeletal muscle and brain health during aging.

## 4 Endocrine and metabolic basis of muscle-brain crosstalk

### 4.1 Skeletal muscle as a hub of inter-organ communication

Skeletal muscle is the largest organ in the non-obese human body, accounting for approximately 40% of total body weight (49). Beyond its mechanical role in converting chemical energy into force, skeletal muscle serves as a major reservoir for energy substrates and plays a central role in metabolic homeostasis (50, 51). These characteristics make skeletal muscle central to movement, energy expenditure, glucose handling, and whole-body metabolic regulation.

With advances in biochemical and molecular research, the endocrine functions of skeletal muscle have become increasingly appreciated. From myokines, exerkines to muscle-derived extracellular vesicles, a diverse array of signaling molecules released by skeletal muscle has been identified. Transported through the circulation to distant tissues, these signaling mediators enable extensive communication between skeletal muscle and multiple organ systems, highlighting the critical role of skeletal muscle as an endocrine organ (52).

Unlike classical endocrine organs that usually release a relatively defined set of hormones, skeletal muscle has a highly dynamic and context-dependent secretory profile (53, 54). This secretome is largely shaped by contractile mode, time and intensity (55). It includes not only classical myokines, but also exerkines, growth factors, metabolites, microRNAs, and muscle-derived extracellular vesicles (56, 57). These factors may act locally through autocrine and paracrine mechanisms or enter the circulation to exert endocrine effects on distant organs. This secretory network allows skeletal muscle to communicate with multiple organs (57, 58), thereby participating in the regulation of systemic metabolism, inflammatory tone, vascular function, tissue remodeling, and adaptation to environmental and physiological stress (53, 56, 59). Importantly, aging and chronic diseases may alter the composition and function of the muscle secretome, shifting it from a protective and adaptive signaling system toward a dysregulated profile characterized by impaired metabolic signaling, increased inflammatory activity, and reduced regenerative capacity (54, 59). Therefore, skeletal muscle should not be viewed merely as a contractile or metabolic tissue, but as an active endocrine organ that contributes to whole-body homeostasis and age-related disease progression.

### 4.2 Bidirectional communication between skeletal muscle and brain

During aging, the brain is increasingly regarded as a key target of skeletal muscle-derived signals (25, 27). For this reason, sarcopenia and cognitive impairment frequently coexist in older adults, and their clinical links are increasingly substantial with age (19, 34). This mutual promotion suggests a bidirectional communication network connecting peripheral muscular status with central nervous system function. In this network, muscle-derived signals may influence neurogenesis, synaptic plasticity, neuroinflammation, brain metabolism, and cognitive function (60, 61). For example, exercise-responsive molecules such as irisin, IGF-1, cathepsin B, BDNF, IL-6, and lactate have been proposed to participate in muscle-to-brain signaling by modulating neuronal survival, hippocampal plasticity, cerebral energy metabolism, and inflammatory balance (28, 62, 63). Muscle-derived extracellular vesicles may also serve as carriers of proteins, lipids, and non-coding RNAs, providing an additional route through which skeletal muscle can deliver regulatory signals to distant tissues, including the central nervous system (57). These findings suggest that skeletal muscle may support brain health not only by enabling movement and physical activity, but also by acting as an endocrine and metabolic signaling organ.

Conversely, the brain plays an essential role in maintaining skeletal muscle function. Central motor networks provide the descending drive required for voluntary activation, and age-related impairments in neural control may contribute to reduced strength and physical performance even when muscle mass loss is modest (64). This central influence is expressed through motor neurons and the neuromuscular junction, where disrupted innervation can weaken motor unit output and accelerate functional decline. Beyond voluntary motor control, the brain also regulates skeletal muscle through autonomic and neuroendocrine pathways. Sympathetic signaling contributes to motor innervation and neuromuscular junction integrity, while dysregulation of the hypothalamic-pituitary-adrenal axis may expose muscle to prolonged glucocorticoid signaling, promoting protein breakdown and suppressing muscle protein synthesis (65, 66). The brain further affects muscle health indirectly through behavior. Cognitive decline may reduce physical activity, weaken exercise adherence, and impair self-management, thereby limiting the behavioral conditions required to preserve skeletal muscle function (67). Thus, brain-to-muscle regulation involves not only motor commands but also autonomic, endocrine, and behavioral mechanisms that may become compromised during aging.

## 5 Disrupted muscle-brain crosstalk in sarcopenia and cognitive impairment

### 5.1 Myokine and exerkine signaling

Skeletal muscle is now recognized as an active secretory organ that releases diverse bioactive molecules, including cytokines, peptides, growth factors, metabolites, and extracellular vesicle-associated signals. Myokines generally refer to muscle-derived signaling molecules, whereas exerkines include a broader range of exercise-responsive factors released by multiple tissues during or after physical activity. Although partly overlapping, the terms are not interchangeable because an exercise-induced change in the circulating concentration of a molecule does not by itself establish skeletal muscle as its principal source (27). Interpretation of circulating mediators also requires caution because tissue-source attribution and assay performance vary across factors. This distinction is particularly relevant when interpreting muscle–brain crosstalk, in which peripheral and central sources may contribute to the same signaling molecule (**Table 1**).

**Table 1:** Selected myokines and exerkines involved in muscle–brain crosstalk.

Factor	Myokine/exerkine classification	Source(s)	Circulating form	Route of brain access/signaling	Cognitive and disease-related relevance
FNDC5/irisin	Myokine and exerkine	Mainly Skeletal muscle	Circulating irisin in plasma	BBB crossing reported, transport mechanism remains unresolved	Synaptic plasticity and memory; beneficial effects reported in AD, post-stroke cognitive impairment, chronic cerebral hypoperfusion/vascular cognitive impairment, and PD-CI
CTSB	Myokine and exerkine	Mainly skeletal muscle	Circulating CTSB; soluble and EVs-associated forms	EVs-mediated across the BBB	Hippocampal neurogenesis and memory; beneficial in AD models but potentially detrimental after ischemic stroke.
IGF-1	exerkine	Mainly liver; also produced in skeletal muscle	Circulating IGF-1, predominantly bound to IGF-binding proteins	LRP1-dependent BBB transport; LRP2/megalin-mediated blood–CSF transport.	Neurogenesis and memory; associated with MCI and vascular dementia risk.
BDNF	exerkine	CNS, skeletal muscle, and other tissues	Circulating BDNF, with substantial platelet-associated storage	Saturable BBB transport; substantial central production.	Neurogenesis, synaptic plasticity, and memory; implicated in MCI and AD

Myokines refer to signaling factors produced and released by skeletal muscle, whereas exerkines refer to factors whose release or activity is modulated by acute or chronic exercise; the two categories may overlap. Routes of brain access or signaling are based on currently available evidence and do not necessarily indicate that a complete peripheral-to-brain pathway has been established in humans. Disease-related effects may vary according to tissue source, disease context, and experimental model. AD, Alzheimer’s disease; BBB, blood–brain barrier; BDNF, brain-derived neurotrophic factor; CNS, central nervous system; CSF, cerebrospinal fluid; CTSB, cathepsin B; EV, extracellular vesicle; FNDC5, fibronectin type III domain-containing protein 5; IGF-1, insulin-like growth factor 1; LRP1, low-density lipoprotein receptor-related protein 1; LRP2, low-density lipoprotein receptor-related protein 2; MCI, mild cognitive impairment; PD-CI, Parkinson’s disease-associated cognitive impairment; VCI, vascular cognitive impairment.

Several exercise-responsive factors have been proposed to participate in communication between skeletal muscle and the brain, including FNDC5/irisin, IGF-1, and cathepsin B (CTSB). BDNF is also closely involved in many of these pathways, but it is more appropriately considered a downstream neurotrophic mediator than a muscle-specific circulating signal (27, 68–70). Current evidence suggests that these molecules act through partially overlapping pathways, and their relevance may vary across different cognitive disorders.

FNDC5/irisin provides one of the most extensively studied experimental examples. Exercise-induced activation of PGC-1α in skeletal muscle can increase FNDC5 expression and irisin release, while experimental studies have linked FNDC5/irisin signaling with hippocampal plasticity and memory. In Alzheimer’s disease mouse models, peripheral FNDC5/irisin overexpression improved synaptic plasticity and memory, whereas blockade of peripheral FNDC5/irisin attenuated the protective effects of exercise (71). More recent evidence extends these findings to other disease contexts: irisin improved cognition in experimental post-stroke and chronic cerebral hypoperfusion models, while a recent clinical-mechanistic study implicated exercise-responsive irisin in Parkinson’s disease-associated cognitive impairment (72–74). Related experimental work also identified a PGC-1α/ERRα/FNDC5 pathway within the brain, through which neuronal FNDC5 regulates hippocampal BDNF expression (70). However, the relative contributions of circulating peripheral irisin and locally expressed neuronal FNDC5 remain unresolved.

IGF-1 illustrates a different form of endocrine–metabolic interaction. Circulating IGF-1 is predominantly liver-derived, although skeletal muscle also produces IGF-1 locally. Blood-borne IGF-1 can enter the CNS through regulated BBB and blood–CSF transport (75, 76), and experimental evidence supports its involvement in exercise-induced hippocampal neurogenesis and BDNF-related responses (77). In humans with mild cognitive impairment, higher serum IGF-1 has also been associated with better global cognition and memory (78). In skeletal muscle, IGF-1 supports protein synthesis, satellite cell activity, and tissue maintenance (79). Thus, IGF-1 represents a systemic growth-factor pathway relevant to both muscle anabolism and brain plasticity rather than a purely muscle-derived signal.

CTSB has also emerged as a candidate exercise-responsive signal. Experimental work identified CTSB as a running-induced muscle-secreted factor, and CTSB deficiency abolished exercise-related increases in hippocampal neurogenesis and spatial memory in mice (69). In humans, exercise-related changes in circulating CTSB were associated with fitness and hippocampus-dependent memory (80). Subsequent mouse studies further showed that muscular OGT/CTSB signaling promotes EV-associated secretion of CTSB and its delivery to the brain, contributing to exercise-induced hippocampal neurogenesis and cognitive improvement, including in APP/PS1 mice (81). Importantly, CTSB effects appear context dependent: in ischemic stroke mice, sustained elevation of brain CTSB was associated with neuronal and cognitive injury, whereas CTSB inhibition improved cognitive outcomes (82). This contrast highlights the importance of tissue source and disease context when interpreting CTSB signaling.

Taken together, current evidence supports several candidate endocrine routes through which muscle activity may influence brain function, but their effects and strength of evidence vary across molecules and disease contexts. Mechanistic support for FNDC5/irisin and CTSB remains largely experimental, whereas IGF-1 involves substantial contributions from non-muscle tissues. Direct demonstration of complete muscle-to-circulation-to-brain signaling sequences in humans remains limited, particularly in older adults with sarcopenia and cognitive impairment.

### 5.2 Inflammation and immune endocrinology

Inflammaging and immune-endocrine dysregulation represent a shared systemic background that may contribute to both sarcopenia and cognitive impairment. Inflammaging refers to a chronic, low-grade systemic inflammatory state that develops with aging and is characterized by persistent activation of innate immune pathways, even without acute infection or injury (83). Unlike transient inflammatory responses that support host defense and tissue adaptation, sustained inflammaging may disrupt metabolic and endocrine homeostasis and increase vulnerability to age-related disorders (84, 85). In muscle-brain crosstalk, inflammaging is therefore better viewed not as a single upstream cause, but as a systemic process that can both contribute to and be reinforced by muscle dysfunction, metabolic disturbance, reduced physical activity, and brain aging (**Figure 2**).

**Figure 2:**
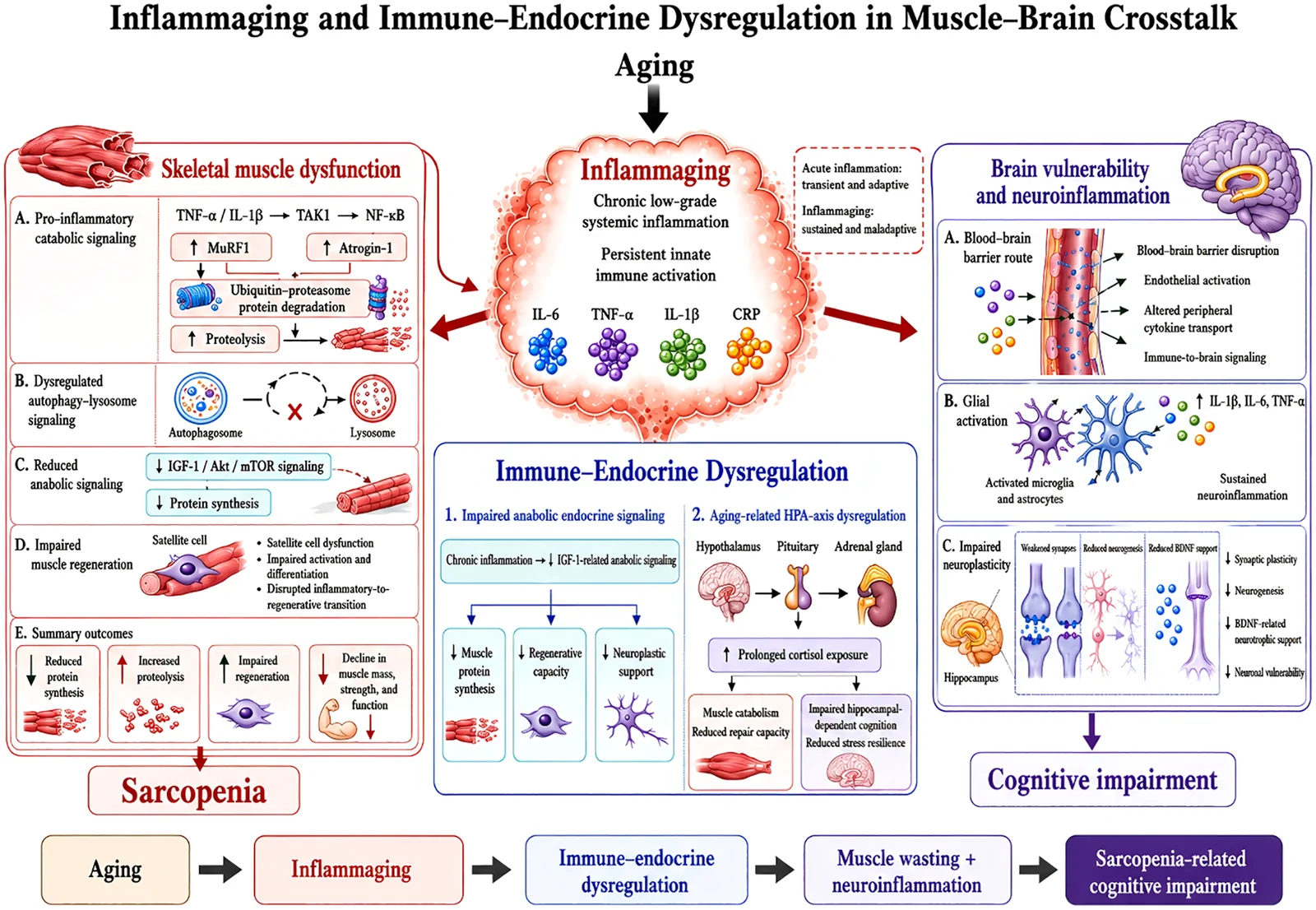
Inflammaging and immune-endocrine dysregulation in muscle-brain crosstalk. IL-6, Interleukin-6; TNF-α, tumor necrosis factor-α; IL-1β, interleukin-1β; CRP, C-reactive protein; TAK1, transforming growth factor-β-activated kinase 1; NF-κB, nuclear factor kappa B; MuRF1, muscle RING-finger protein-1; IGF-1, insulin-like growth factor 1; PKB/Akt, protein kinase B; mTOR, mechanistic target of rapamycin; BDNF, brain-derived neurotrophic factor; HPA axis, hypothalamic–pituitary–adrenal axis.

In skeletal muscle, human studies have consistently associated a higher inflammatory burden with adverse muscle phenotypes. Higher circulating concentrations of inflammatory cytokines, including IL-6 and TNF-α, together with inflammatory markers such as CRP, have been associated with lower muscle mass, reduced strength, and poorer physical performance in older adults (86, 87). Experimental studies provide potential mechanisms for these associations. At the cellular level, sustained TNF-α- and IL-1β-related signaling can activate TAK1 and NF-κB, increasing the expression of atrophy-related genes such as MuRF1 and atrogin-1 (88). These E3 ubiquitin ligases promote ubiquitin-proteasome-mediated protein degradation, a major proteolytic route in skeletal muscle atrophy (89). Inflammatory signaling may also dysregulate autophagy-lysosome pathways, counteract IGF-1/Akt/mTOR-mediated protein synthesis, alter FoxO-dependent proteolytic control, and impair satellite cell activation, differentiation, and regenerative transition (79, 90, 91). These processes favor muscle catabolism over repair, thereby accelerating the decline in muscle quality and function.

A systemic inflammatory environment may compromise brain health through several converging routes. Experimental and translational studies indicate that peripheral inflammatory signals can influence the central nervous system through the cerebrovascular endothelium, cytokine transport mechanisms, and changes in blood–brain barrier integrity (92, 93). Age-related impairment of the blood–brain barrier may further increase brain vulnerability to peripheral inflammatory signals (94). Within the CNS, activation of microglia and astrocytes can increase the production of cytokines such as IL-1β, IL-6, and TNF-α, which may contribute to the persistence of neuroinflammatory signaling (95). This inflammatory environment may weaken synaptic plasticity and neurotrophic support, thereby increasing neuronal vulnerability. Epidemiological evidence is broadly consistent with this model, showing elevated inflammatory markers in MCI and Alzheimer’s disease and an association between higher circulating IL-6 and subsequent cognitive decline (92). These observations, however, do not establish a direct causal pathway from peripheral inflammation to cognitive impairment.

Immune-endocrine dysregulation may further interact within this inflammatory environment. In skeletal muscle, persistent inflammation may weaken IGF-1-related maintenance and repair pathways, reducing protein synthesis and regenerative capacity. Meanwhile, aging-related hypothalamic-pituitary-adrenal (HPA) axis dysregulation may lead to prolonged cortisol exposure, promoting muscle catabolism while impairing hippocampal-dependent cognition and stress resilience (96–98). Thus, chronic inflammation should not be regarded merely as an immune disturbance, but as a process integrated with endocrine and metabolic regulation. These interactions create a shared biological environment in which skeletal muscle becomes less capable of regeneration, while the brain becomes more vulnerable to neuroinflammatory and neuroendocrine stress, thereby reinforcing the coexistence of sarcopenia and cognitive impairment.

Importantly, the biological effects of inflammatory mediators depend on their temporal and physiological context. IL-6 provides a particularly relevant example. Chronic elevations in circulating IL-6 during aging are associated with systemic inflammatory burden and adverse muscle and cognitive outcomes, whereas acute exercise induces a transient increase in muscle-derived IL-6 that participates in metabolic and adaptive signaling (27). The effects attributed to TNF-α in this review similarly refer mainly to sustained elevations and chronic inflammatory signaling. These responses should not be regarded as biologically equivalent. Thus, chronic pathological inflammation should be distinguished from transient exercise-induced cytokine signaling when interpreting inflammation in muscle–brain crosstalk.

Taken together, current evidence supports chronic immune-inflammatory dysregulation as a plausible shared contributor to muscle and brain vulnerability during aging. Human studies primarily demonstrate associations between inflammatory burden, sarcopenia-related phenotypes, and cognitive outcomes, whereas many of the proposed cellular and molecular pathways are supported predominantly by experimental evidence. A complete causal sequence linking peripheral inflammation, skeletal muscle degeneration, central neuroinflammation, and cognitive impairment has not yet been established in humans. Inflammaging may therefore act as a systemic process that increases vulnerability in both skeletal muscle and the brain and reinforces age-related dysfunction.

### 5.3 Mitochondria and oxidative stress

Skeletal muscle and the brain are both highly energy-demanding tissues that rely on efficient mitochondrial function to sustain contraction, synaptic activity, and metabolic adaptation. During aging, mitochondrial biogenesis declines, oxidative phosphorylation capacity decreases, and mitochondrial quality control is progressively impaired. These changes increase mitochondrial reactive oxygen species (mtROS) production and contribute to both skeletal muscle decline and brain aging (99–102) (**Figure 3**). These abnormalities are observed in both skeletal muscle and the aging brain, suggesting that mitochondrial dysfunction represents a shared biological feature of sarcopenia and cognitive impairment.

**Figure 3:**
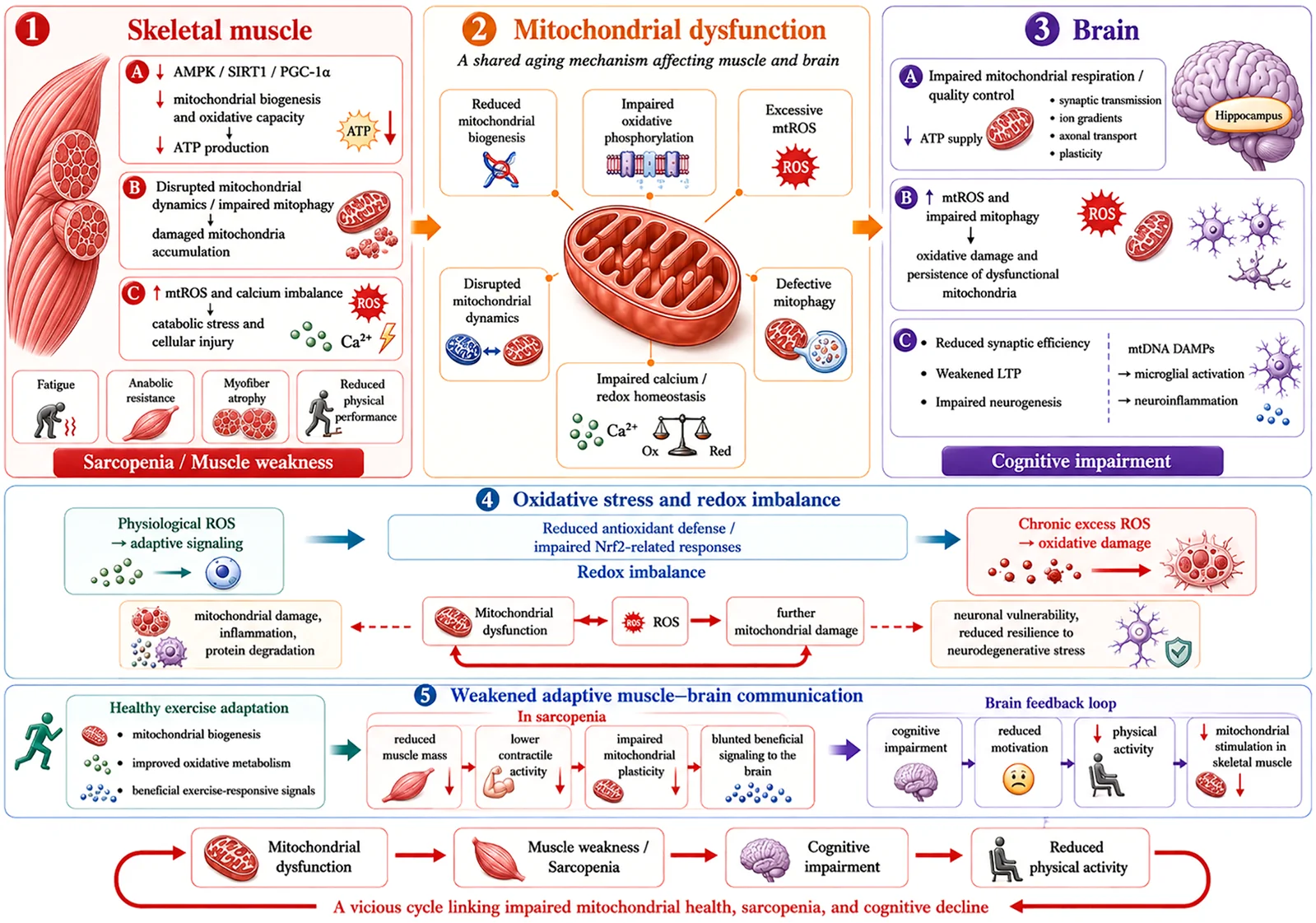
Mitochondrial dysfunction and oxidative stress in muscle-brain crosstalk. AMPK, Adenosine monophosphate-activated protein kinase; SIRT1, sirtuin 1; PGC-1α, peroxisome proliferator-activated receptor γ coactivator 1α; ATP, adenosine triphosphate; ROS, reactive oxygen species; mtROS, mitochondrial reactive oxygen species; Ca²^+^, calcium ion; NF-κB, nuclear factor kappa B; Nrf2, nuclear factor erythroid 2-related factor 2; GSH, glutathione; GSSG, glutathione disulfide; DAMPs, danger-associated molecular patterns; DNA, deoxyribonucleic acid.

In skeletal muscle, mechanistic studies indicate that aging-related mitochondrial impairment can impair energy metabolism and contractile function. Reduced AMPK/SIRT1/PGC-1α-related signaling may weaken mitochondrial biogenesis and oxidative capacity, limiting ATP production during contraction (103, 104). Meanwhile, disrupted fusion-fission balance and impaired mitophagy further promote the accumulation of damaged mitochondria, increasing mtROS generation and reducing metabolic efficiency (105, 106). Excessive mtROS can damage mitochondrial components, disturb calcium homeostasis, and activate catabolic stress pathways. As a result, skeletal muscle becomes more vulnerable to fatigue, anabolic resistance, myofiber atrophy, and functional decline. However, their relative contribution to human sarcopenia remains incompletely defined.

In the brain, experimental and disease-model evidence similarly links impaired mitochondrial respiration and quality control with reduced neuronal energy availability, oxidative injury, and synaptic dysfunction, particularly in metabolically vulnerable regions such as the hippocampus (107, 108). Mitochondrial damage may also amplify neuroinflammatory signaling through mtROS and mitochondrial danger-associated molecular patterns. Importantly, ROS effects are context dependent: transient ROS production can contribute to exercise adaptation, whereas sustained oxidative stress may promote cellular damage. Age-related impairment of antioxidant responses, including Nrf2-related signaling, may further increase vulnerability in both muscle and brain (109).

Mitochondrial dysfunction may also influence muscle–brain communication by altering the metabolic and endocrine responses generated by contracting skeletal muscle. In sarcopenia, reduced contractile activity and mitochondrial plasticity may attenuate these adaptive signals, whereas cognitive impairment and lower physical activity may further reduce mitochondrial stimulation in muscle. Such interactions suggest a potentially reinforcing relationship among mitochondrial dysfunction, physical inactivity, muscle decline, and cognitive deterioration. However, direct evidence for a complete muscle-to-brain mitochondrial pathway in humans remains limited. Current evidence more strongly supports mitochondrial dysfunction and oxidative stress as shared and interacting processes that may contribute to impaired muscle–brain crosstalk during aging.

### 5.4 Insulin resistance and glucose metabolism

Skeletal muscle is the major site of insulin-stimulated glucose disposal and plays a central role in whole-body glucose homeostasis. Through IRS-1/PI3K/Akt signaling, insulin promotes GLUT4 translocation and glucose uptake, while Akt/mTOR-related signaling supports muscle protein synthesis. During aging, muscle loss, reduced contractile activity, intramuscular lipid accumulation, mitochondrial dysfunction, and chronic inflammation may impair skeletal muscle insulin sensitivity. Human evidence links these abnormalities with reduced glucose disposal, impaired anabolic signaling, and sarcopenic phenotypes (110, 111).

In skeletal muscle, insulin resistance promotes sarcopenia through several interconnected mechanisms. In obese mice, Li et al. found that impaired Akt signaling reduces mTOR-mediated protein synthesis and weakens inhibition of FoxO transcription factors, increasing atrophy-related gene expression and proteolysis (112). Furthermore, reduced insulin-stimulated glucose uptake may also limit metabolic support for contraction and repair, whereas intramyocellular lipid accumulation may aggravate lipotoxicity and local inflammation (113–115). These findings provide a plausible basis for a reciprocal relationship between impaired muscle metabolism and systemic insulin resistance (**Figure 4**).

**Figure 4:**
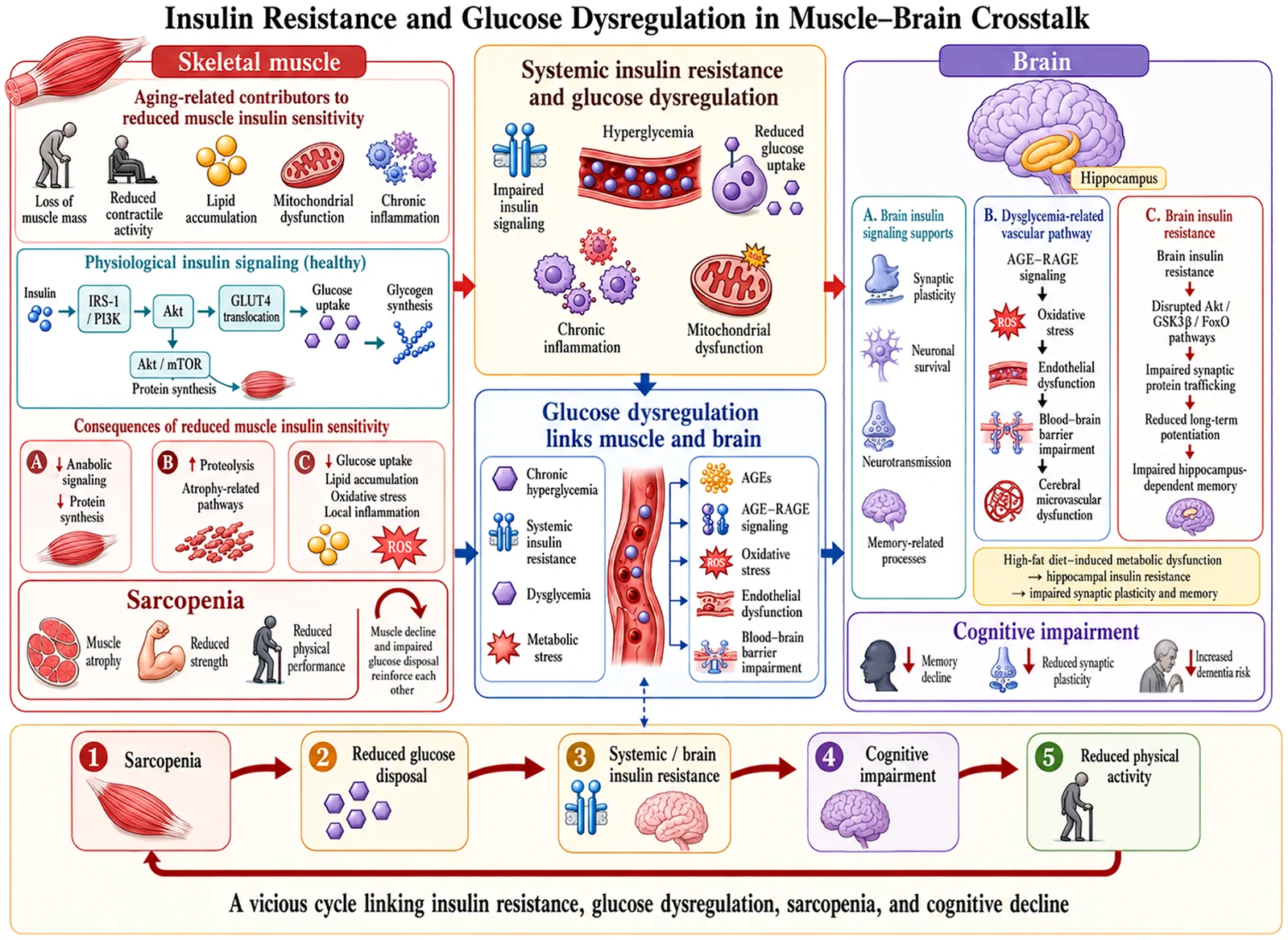
Insulin resistance and glucose dysregulation in muscle-brain crosstalk. IRS-1, Insulin receptor substrate 1; PI3K, phosphoinositide 3-kinase; PKB/Akt, protein kinase B; GLUT4, glucose transporter type 4; mTOR, mechanistic target of rapamycin; FoxO, forkhead box O; AGEs, advanced glycation end-products; RAGE, receptor for advanced glycation end-products; ROS, reactive oxygen species; GSK3β, glycogen synthase kinase-3β; BBB, blood-brain barrier.

In humans, systemic insulin resistance and chronic hyperglycemia have been associated with poorer cognitive outcomes and an increased risk of cognitive impairment and dementia (116). Several mechanisms may contribute to these associations. Glucose dysregulation may affect the brain through vascular and metabolic pathways, including disruption of blood–brain barrier integrity and activation of the advanced glycation end product–receptor for advanced glycation end product (AGE–RAGE) axis, both of which have been implicated in diabetes-related cognitive dysfunction (117, 118). Brain insulin signaling provides another potential pathway. Insulin receptors are widely expressed in cognition-related regions, including the hippocampus, where insulin signaling contributes to synaptic plasticity, neuronal survival, neurotransmission, and memory processes (119). Experimental models further indicate that impaired brain insulin signaling can disrupt Akt/GSK3β/FoxO-related pathways and hippocampal synaptic function (120)|.

Together, insulin resistance and glucose dysregulation represent a shared metabolic disturbance that may link impaired skeletal muscle metabolism with increased metabolic vulnerability in the brain (**Figure 4**).

### 5.5 Lactate-mediated metabolic signaling

Lactate represents a distinctive metabolic component of muscle–brain crosstalk because it acts not only as an energy substrate but also as an exercise-responsive signaling molecule. During muscle contraction, skeletal muscle produces and releases lactate into the circulation. Circulating lactate can cross the blood-brain barrier through monocarboxylate transporters and enter neural and glial cells. In the brain, lactate may function as either an oxidative substrate or a signaling mediator, thereby linking peripheral muscle activity with central nervous system adaptation (**Figure 5**).

**Figure 5:**
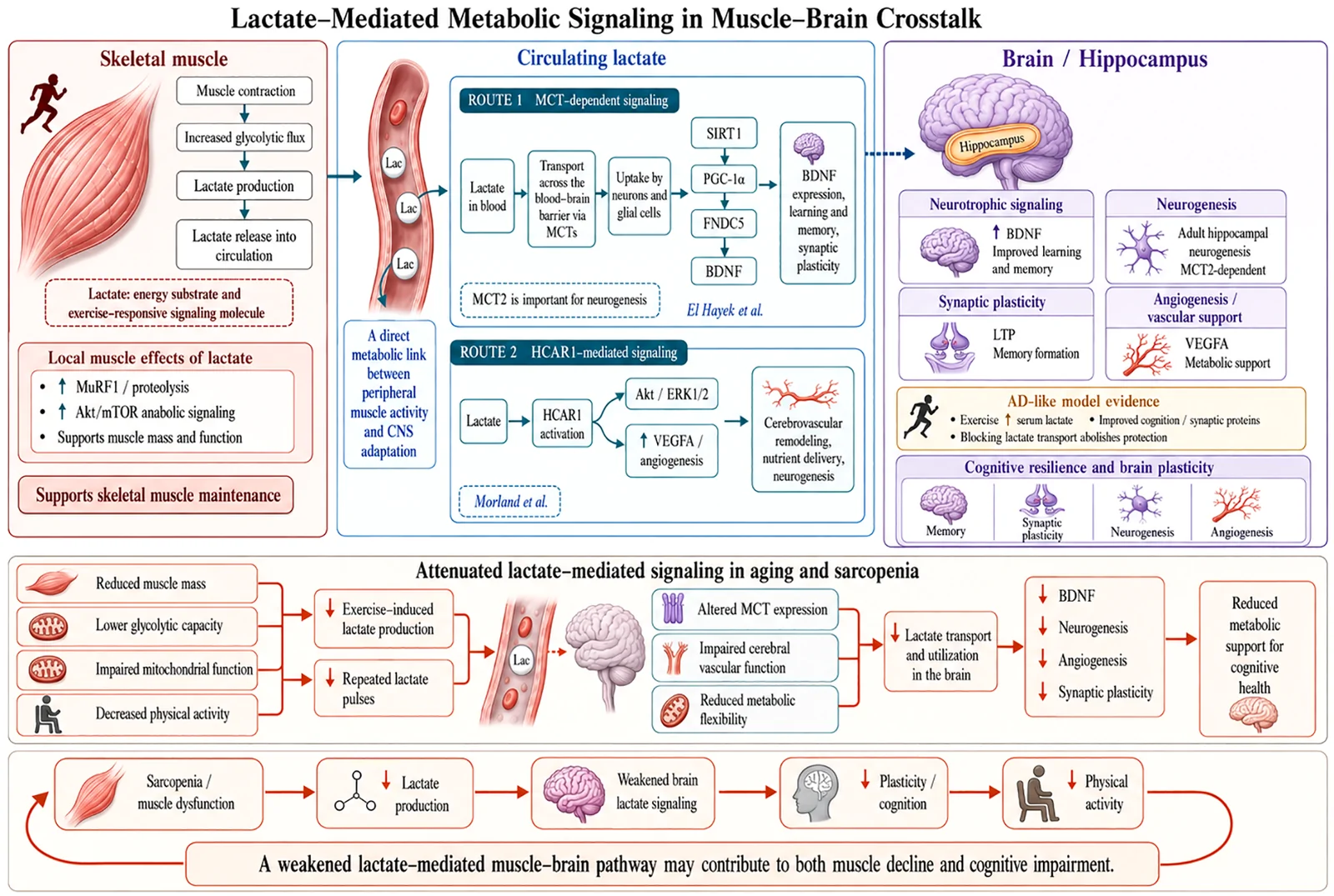
Lactate-mediated metabolic signaling in muscle-brain crosstalk. AD, Alzheimer’s disease; Akt, protein kinase B; BDNF, brain-derived neurotrophic factor; CNS, central nervous system; ERK1/2, extracellular signal-regulated kinase 1/2; FNDC5, fibronectin type III domain-containing protein 5, HCAR1, hydroxycarboxylic acid receptor 1; Lac, lactate; LTP, long-term potentiation; MCT, monocarboxylate transporter; MCT2, monocarboxylate transporter 2; mTOR, mechanistic target of rapamycin; MuRF1, muscle RING-finger protein 1; PGC-1α, peroxisome proliferator-activated receptor gamma coactivator 1-alpha; SIRT1, sirtuin 1; VEGFA, vascular endothelial growth factor A.

Experimental studies support a role for lactate in exercise-related brain plasticity. In mice, exercise-related lactate signaling increased hippocampal Bdnf expression through SIRT1 and the PGC-1α/FNDC5 pathway and was associated with improved learning and memory (121). Lactate can also signal through hydroxycarboxylic acid receptor 1 (HCAR1). High-intensity exercise or lactate administration increased cerebral VEGFA expression and angiogenesis in wild-type mice, whereas these responses were attenuated in Hcar1-deficient animals (122). These findings suggest that lactate may influence the brain through both transporter-dependent uptake and receptor-mediated signaling.

Additional experimental evidence highlights the importance of lactate availability and transport within the brain. Systemic L-lactate administration promoted adult hippocampal neurogenesis in an MCT2-dependent manner (123), while astrocyte-derived lactate and astrocyte–neuron lactate transport have been shown to support long-term memory formation and synaptic plasticity (124). However, the latter findings primarily concern local brain lactate metabolism and should not be interpreted as direct evidence for a skeletal muscle-to-brain pathway. More recently, exercise-induced increases in circulating lactate have been linked experimentally to improved cognition and synaptic outcomes in Alzheimer’s disease-like mouse models (125), further supporting lactate as a candidate mediator of exercise-related brain adaptation.

Lactate may also influence skeletal muscle homeostasis. Animal studies suggest that lactate can attenuate muscle atrophy and modulate pathways related to protein degradation and anabolic signaling, including MuRF1 and Akt/mTOR-related signaling (126, 127). These findings raise the possibility that lactate participates in adaptive processes relevant to both muscle maintenance and brain plasticity. In aging and sarcopenia, reduced muscle mass and contractile activity could diminish exercise-induced lactate responses, potentially weakening this form of metabolic communication. However, direct evidence that impaired lactate signaling contributes to cognitive impairment in older adults with sarcopenia remains limited, and this proposed pathway is supported predominantly by experimental studies based on animal models.

## 6 Muscle–brain crosstalk modulation by exercise

Exercise provides a physiological means of modulating the endocrine and metabolic pathways involved in muscle–brain crosstalk. Beyond improvements in muscle strength and physical performance, exercise may alter the biological environment through which skeletal muscle and the brain communicate. This makes exercise particularly relevant to the inter-organ crosstalk that may be disrupted in sarcopenia and cognitive impairment. Importantly, the value of exercise in this context does not depend on a single signaling pathway. Rather, exercise provides a dynamic physiological stimulus through which several components of muscle–brain communication can be modified simultaneously.

As a core exercise modality recommended for the management of sarcopenia, resistance exercise provides a useful example of how adaptations within skeletal muscle may be accompanied by systemic signals with potential relevance to the brain. In older adults, resistance training can partly restore impaired anabolic responsiveness and produce substantial gains in muscle strength. These adaptations are accompanied by changes in circulating signals, with IGF-1 being particularly typical. Systematic evidence from 1,247 middle-aged and older adults indicates that resistance exercise increases circulating IGF-1, but not BDNF, reporting a greater role for IGF-1 in attenuating age-related neurological and cognitive decline (128). As mentioned earlier, skeletal muscle is the main site where insulin stimulates glucose metabolism. The promoting effect of resistance exercise on skeletal muscle is also reflected in insulin sensitivity, which has also been widely reported in the elderly (129). Such changes are relevant because impaired insulin action and reduced anabolic signaling are involved in both muscle deterioration and brain vulnerability. Clinical findings provide a further link with cognition. Among older adults with mild cognitive impairment, cognitive improvement after resistance training was partly mediated by gains in strength (130).

Sustained metabolic demand during aerobic exercise provides a different context in which muscle–brain communication may be modified. Human skeletal muscle studies show that aerobic training can enhance mitochondrial biogenesis and mitochondrial quality control in older adults (131). This is relevant to the mitochondrial dysfunction described earlier in both sarcopenia and cognitive impairment. Evidence from older adults indicates that aerobic exercise may influence muscle–brain crosstalk through distinct systemic responses, including reductions in TNF-α-related inflammatory signaling and increases in circulating CTSB (80, 132). These responses suggest that aerobic exercise may influence the biological environment shared by muscle and brain through both reduced inflammatory signaling and enhanced exercise-responsive myokine signaling. Aerobic training may also produce measurable adaptations in the brain. In older adults, changes in hippocampal volume and memory following aerobic exercise have been linked to circulating BDNF (133).

Across exercise modalities, lactate is a common metabolic response to muscular activity, and the magnitude of the lactate response depends more on metabolic demand, including exercise intensity, duration, and recovery characteristics. This makes lactate a particularly relevant cross-modal candidate for muscle–brain signaling. Rather than acting solely as a metabolic by-product, lactate generated from skeletal muscle can cross the blood–brain barrier and participate in brain signaling. Experimental evidence converges on the PGC-1α/FNDC5–BDNF axis as one pathway involved in this response. Exercise has been linked to hippocampal BDNF induction through PGC-1α/FNDC5 signaling (70), while subsequent work showed that lactate can act upstream of this pathway. In mice, exercise-derived lactate activated SIRT1 and increased PGC-1α and FNDC5, leading to enhanced hippocampal BDNF/TRKB signaling and improved spatial learning and memory (121). Lactate may also influence the brain through HCAR1-dependent signaling, which has been linked to cerebral VEGFA expression and angiogenesis (122). Together, these findings position lactate as a plausible metabolic mediator linking exercise-induced peripheral activity with brain plasticity. However, this mechanistic evidence remains largely preclinical, and the same signaling sequence has not been directly established in humans.

This lactate-centered mechanism also provides a rationale for considering exercise strategies that intensify metabolic stress. Blood flow restriction (BFR) is particularly relevant in this context because it can increase metabolite accumulation during exercise while maintaining a relatively low mechanical load (134). Acute BFR exercise produces marked local metabolic stress and lactate accumulation, creating a physiological setting in which lactate-related muscle–brain signaling may be enhanced. Preliminary cognitive findings are consistent with this possibility. BFR-based exercise has been associated with changes in circulating BDNF and cognitive outcomes in older adults (135). Increased lactate has also been associated with improved interference control after acute BFR exercise, although mediation analysis did not support lactate as the direct mediator of this effect (136). Thus, BFR may provide a useful experimental model for examining metabolically driven muscle–brain communication, but evidence that amplified lactate signaling explains its cognitive effects remains preliminary.

Taken together, current exercise evidence supports the modifiability of muscle–brain crosstalk. The magnitude and pattern of endocrine and metabolic responses may differ across exercise modalities (e.g., BDNF), while several signaling processes are shared across them (e.g., lactate). Human studies usually capture only part of the proposed signaling chain. A trial may identify changes in muscle function or circulating biomarkers together with cognitive improvement, yet rarely establishes the muscle origin of a mediator and its subsequent action within the brain. This limitation is particularly important because sarcopenia and cognitive impairment are usually studied separately. Evidence in individuals with both conditions remains sparse. Exercise should therefore be regarded as a physiologically relevant context in which endocrine–metabolic muscle–brain communication can be investigated and potentially modified, rather than as proof that any single pathway explains the coexistence of sarcopenia and cognitive impairment. Future studies should directly test these pathways in populations with combined muscle and cognitive vulnerability. Designs that connect peripheral biological responses with brain outcomes will be particularly important.

## 7 Conclusions

This review highlights muscle–brain crosstalk as a useful framework for understanding the coexistence of sarcopenia and cognitive impairment. Myokine and exerkine signaling, inflammaging and immune endocrinology, mitochondria and oxidative stress, insulin resistance and glucose metabolism, and lactate-mediated metabolic signaling represent potential pathways involved in muscle–brain crosstalk. However, the strength of evidence differs across these mechanisms, and many proposed inter-organ pathways remain supported primarily by experimental rather than direct human evidence.

Exercise provides a dynamic physiological context for modulating and investigating muscle–brain crosstalk, with both shared and modality-specific endocrine and metabolic responses. Future studies should clarify the temporal and cross-pathway relationships underlying muscle–brain crosstalk in humans. In individuals with coexisting sarcopenia and cognitive impairment, clinical translation will require integrating muscle and cognitive phenotypes with circulating biomarkers, metabolic measures, and neuroimaging.
